# Violence against physicians in Jordan: An analytical cross-sectional study

**DOI:** 10.1371/journal.pone.0245192

**Published:** 2021-01-25

**Authors:** Ruba Alhamad, Aiman Suleiman, Isam Bsisu, Abeer Santarisi, Ahmad Al Owaidat, Albatool Sabri, Mohammad Farraj, Mohammad Al Omar, Rawan Almazaydeh, Ghada Odeh, Mohammad Al mousa, Mohamad Mahseeri

**Affiliations:** 1 Department of Emergency Medicine and Accidents, University of Jordan, School of Medicine, Amman, Jordan; 2 Department of Anesthesia and Intensive Care, University of Jordan, School of Medicine, Amman, Jordan; 3 Department of General Surgery, University of Jordan, School of Medicine, Amman, Jordan; 4 Department of Oral and Maxillofacial Surgery, University of Jordan, School of Medicine, Amman, Jordan; Maragheh University of Medical Sciences, ISLAMIC REPUBLIC OF IRAN

## Abstract

**Background:**

High numbers of violence incidents against physicians are reported annually in both developing and developed countries. In Jordan, studies conducted on healthcare workers involved small number of physicians and showed higher percentages of violence exposure when compared to other investigations from the Middle East. This is a large study aiming to comprehensively analyze the phenomenon in the physicians’ community to optimize future strategies countering it.

**Methods:**

The study has a cross sectional, questionnaire-based design. It targeted 969 doctors from different types of healthcare Jordanian institutions in Amman, between May to July, 2019. The questionnaire was designed to evaluate properties of reported abuse cases in terms of abusers, timing, and type of abuse, in addition to the consequences of this abuse.

**Results:**

Prevalence of exposure to violence in the last year among doctors was 63.1% (611 doctors). 423 (67.2%) of male doctors had an experience of being abused during the last 12 months, compared to 188 (55.3%) of females (p< 0.001). Governmental centers showed the highest prevalence. Among 356 doctors working in governmental medical centers, 268 (75.3%) reported being abused (p< 0.001), and they were more abused verbally (63.5%) and physically (10.4%) compared to other medical sectors (p <0.001). The mean score of how worried doctors are regarding violence at their workplace from 1 to 5 was 3.1 ± 1.3, and only 129 (13.3%) believed that they are protected by law.

**Conclusions:**

The study emphasized on the higher rate of violence against physicians in the governmental sector, in addition to the negative effect of abuse on their performance. Moreover, male physicians had higher incidence of workplace abuse. Therefore, strategies that ease and promote the real application of anti-violence policies should become our future target.

## Introduction

Violence against physicians is a growing problem in both developing and developed countries [1]. According to World Health Organization (WHO), 8% to 38% of healthcare providers worldwide might suffer from violence at least once in their career lifetime [2].The National Institute for Occupational Safety and Health (NIOSH) gave a definition for occupational violence as ‘any violent act, including physical assaults or threats of assaults, that is directed towards people at work or on duty’ [3]. The source of violence can be the patient, his/her own relatives, or even coworkers [4]. Violence can be verbal, physical, emotional or even sexual [5,6]. In rare instances, violence can come in the form of grievous hurt or murder [7].

Studies analyzing the factors related to violence against physicians are widely adopted to build strategies that efficiently encounter this phenomenon and update them according to outcomes. Many studies conducted in countries near to Jordan were utilized to build guidelines and protocols that protect healthcare workers. Definitely, workplace violence against physicians is considered a significant issue, with 78.1% reporting being subjected to violence in a cross-sectional survey conducted in Turkey, and 65.9% reporting more than one incident [8]. In Saudi Arabia, two studies were conducted on healthcare workers in 2009 and 2018, with violence exposure prevalences of 28% and 57.5%, respectively [9,10]. Prevalences recorded in the Levant region of the Middle East were higher, as two studies conducted in Palestinian hospitals in 2012 and 2013 showed prevalences of 80.4% and 71.2%, respectively [11,12]. Another study conducted in Lebanon in 2015 on 915 nurses found that 62% were exposed to verbal abuse, and 10% were exposed to physical abuse [13]. In Egypt, a study conducted in the emergency department of Ismailia hospital, found that 59.7% of healthcare workers have suffered from verbal and physical abuse, of which only 23.8% reported the abuse [14]. With the increase in the incidence of violence acts against doctors in the recent years, concerns regarding the decrease in the efficiency of health care delivery led to partial strikes in Egypt in 2012 by medical syndicates asking for an increase in security at healthcare establishments [15]. In Jordan, there were three studies involving mainly the nursing community, which showed an alarming increase in the prevalence of violence against nurses in Jordan from 68.2% in 2003 to 91.4% in 2015 [16–18].

This study is a large study to analyze the issue from physicians’ perspective in Jordan. Health care policies regarding staff protection in Jordan are renewed on yearly bases to adapt the results of new studies concerning occupational violence. The aim of the study is to explore this phenomenon in the physicians’ community, analyze the properties of reported abuse cases in terms of abusers, timing, type of abuse and its consequences, and seek out an effective role-based approach to counter the growing problem. Physicians are involved in the multidisciplinary team approach towards patients in a way that differs from that of nurses, which might help to analyze the violence towards healthcare providers from a different aspect.

## Methodology

### Study design

This is a cross-sectional study conducted on a random sample of medical doctors working in public, private, university and military hospitals. It included doctors from all levels of postgraduate programs: interns, general practitioners, residents, specialists and consultants. All included doctors were required to have working experience equal or more than 6 months at their current medical sector. The study and its questionnaire were approved by the Institutional Review Board (IRB) at the University of Jordan (reference number: 67/2020/823).

According to Jordan Medical Association (JMA), there are 28000 registered physicians in Jordan [19]. The hypothesized prevalence of violence against physicians was 60% [13,14]. Therefore, within a 99% confidence level, a z-score of 2.58, and a 5% margin of error, the study population was determined to be 651 using the formula where sample size = (Z^2^*p*(1-p)/e^2^)/(1 + (Z^2^*p*(1-p))/(e^2^*N)); where N = population size, e = margin of error (percentage in decimal form), and Z = z-score. (https://www.surveymonkey.com/mp/sample-size-calculator/) [8]. We further increased the sample size of the study to decrease the margin of error. The original sample included 1021 doctors, of which 52 surveys were excluded during data validation process, due to lack informative explanations or presence of contradictions in the survey. Our final sample included 969 doctors.

### Questionnaire

Our survey instrument was a questionnaire that was designed to evaluate different related aspects to violence phenomenon. Violence was defined as incidents where physicians are abused, threatened or assaulted in settings related to their work, which can be considered a challenge to their safety, well-being or health [20]. These aspects included type of violence, frequency, in addition to relationships among violence, feelings of safety and satisfaction, which were stratified on the bases of multiple previous studies and were structurally formulated by Gates et al [21]. The questionnaire was web-based, and filled using Google forms [**S1 File**]. We collected data related to four main aspects: demographics of studied participants, properties of the reported abuse cases in terms of abusers, timing, and type of abuse, consequences of abuse on the abused participants and opinions of participants about the phenomenon. We included questions about different forms of violence, assigning incidents to four main categories: verbal, physical, emotional and sexual [22]. Verbal violence was meant to include any type of verbal insult that does not involve physical contact, sexual allusions or emotional abuse. Emotional violence was defined as the indirect exposure of the doctor to a behaviour that resulted in depression or posttraumatic stress disorder. Finally, physical violence was defined as any form of aggressive physical contact or uncomfortable physical behaviour towards the doctor. Moreover, we explore how worried doctors are regarding violence at their workplace from 1 to 5, where 1 signifies “not worried” and 5 signifies “very worried.”

### Data collection and analysis

Data was collected in the months May to July, 2019. Questionnaire and written consent form were sampled using Google forms web-based questionnaire, which was sent to eligible physicians on individual basis by e-mail, and email receival was confirmed by phone. The consent was filled on the first page of the online questionnaire, prior to commencing the questionnaire. The signature was done by checking “I agree” on that page, before proceeding to the questionnaire. Questionnaire was filled by every consented doctor afterwards. Of the 1988 doctors to whom questionnaires were sent, 1021 physicians filled it, for which the response rate was 51.4%. Data was analysed using Statistical Package for Social Science program (SPSS) version 23.0 (SPSS Inc., Chicago, IL, USA) [23]. The data were analysed using the Pearson Chi-squared (χ^2^) test and Fisher's exact test for categorical variables. Independent sample t-test was used to explore how worried doctors are regarding violence at their workplace, using which we investigated for statistically significant difference regarding gender, as well as history of previous injuries related to abuse incidents, in terms of worrying levels. Moreover, Pearson's correlation coefficient (Pearson's r) was used to examine the relationship between the aforementioned score and age. Moreover, we applied multivariable regression analysis to investigate for predictors of abuse in the last 12 months. The statistical significance level was considered as a p-value less than 0.05.

## Results

Overall, 969 participants were included in our study, of which 629 (64.9%) were males and 340 (35.1%) were females. Their mean age was 30.1 ± 6.4 years. Of the 969 included doctors, 356 (36.7%) were working in governmental medical centers, 276 (28.5%) were working in university hospitals, 198 (20.4%) in private centers, and 139 (14.3%) in military hospitals [**Table 1**]. The properties of the reported abuse cases in terms of type of abuse, abusers, and timing are discussed in **Table 2**, and the consequences of abuse on participants was explored in **Table 3**.

**Table 1:** Demographics of studied participants.

Characteristic		Number (n)	Percent (%)
**Gender**	Female	340	35.1
	Male	629	64.9
**Job description**	Consultant	43	4.4
	General practitioner	204	21.1
	Intern	167	17.2
	Resident	439	45.3
	Specialist	116	12.0
**Workplace (sector)**	Government	356	36.7
	Military	139	14.3
	Private	198	20.4
	University/teaching	276	28.5
**Nationality**	Jordanian	924	95.4
	Non-Jordanian	45	4.6
**Work in shifts**		664	68.5

**Table 2:** Properties of the reported abuse cases in terms of abusers, timing, and type of abuse.

Characteristic		Frequency (n)	Percent (%)
**Abuse in the last 12 months**	No	358	36.9
	Yes	611	63.1
**Overall frequency of abuse**	Once	186	19.2
	2–3	251	25.9
	more than 3	210	21.7
	Not specified	322	33.2
**Properties of the abuse and abusers**			
**Type of abuse**	Verbal	545	56.2
	Physical	54	5.6
	Emotional	58	6.0
	Sexual	4	0.4
	Not specified	308	31.8
**Weapon used by abuser**		6	0.6
**The abuser**	The patient	144	14.9
	Relatives of patient	433	44.7
	Co-worker	42	4.3
	Not specified	350	36.1
**Reported the abuse**		128	13.2
**Response to abuse**	Inform a legal party about the abuse	53	5.5
	Tried to defend myself verbally	98	10.1
	Tried to defend myself physically	44	4.5
	Took no action	443	45.7
	Not specified	331	34.2
**Timing**			
**Specific Shift**	Day shift	350	36.1
	Night shift	296	30.5
	Not specified	323	33.3
**Day of the week**	Weekdays	454	46.9
	Weekdays and weekend	67	6.9
	Weekend	116	12.0
	Not specified	332	34.3
**Injured from the attack**		70	7.2
**Emotions experienced as a consequence of violence**	Headache and fatigue	177	18.3
	Fear or stress	280	28.9
	Anger or frustration	454	46.9
	Sadness	248	25.6
	Irritability	256	26.4
	difficulty sleeping	98	10.1
	Suicidal thoughts	2	0.2
	Being a victim of racism	2	0.2
**Family or friends have been threatened because of violence**		190.0	19.6

**Table 3:** Consequences of abuse on doctors.

Characteristic		Frequency (n)	Percent (%)
**Required formal treatment**	No treatment	531	54.8
	Self-treatment	86	8.9
	Physical (treated for an injury)	14	1.4
	Psychiatric	19	2.0
	Not specified	319	32.9
**Job performance affected**		696	71.8
**Feel unsafe at workplace**		677	69.9
**Work changes did occur as a result of violence**	Leave or absence	92	9.5
	Transfer to another location	92	9.5
	Lost their job	12	1.2
	Nothing	773	79.8

Overall, 63.1% of participants (611 doctors) were exposed to violence. We found a significant relationship between gender and the physician being abused, since 423 (67.2%) of males had an experience of being abused during the last 12 months, compared to 188 (55.3%) of females (p< 0.001), and female doctors were less attacked during night shifts 70 (20.6%) when compared with male doctors 226 (35.9%) (p < 0.001). Furthermore, more males were victims of verbal 374 (59.5%) compared to females 171 (50.3%) (p = 0.006), and physical abuse followed the same trend, with 51 (8.1%) of males, compared to 3 (0.9%) of females being physically abused (p< 0.001). No significant difference was found between genders regarding verbal abuse, while sexual abuse was confined to females, since the 4 sexual abused doctors were females (p = 0.006). Moreover, 5 (0.8%) of males were attacked using a weapon, compared to only 1 (0.3%) female (p< 0.001), and overall, 57 (9.1% of males were injured, compared to 13 (3.8%) of females (p = 0.002). Interestingly, upon investigating their point of view, males tend to believe that male doctors are more prone to abuse (305; 48.5%), compared to females (52; 8.3%), while the remaining 272 (43.2%) believed there were no difference (p< 0.001), and only 86 (13.7%) of them were satisfied with current local policy at their institution, compared to 68 (20%) of females (p = 0.01).

Of the 356 working in governmental medical centers, 268 (75.3%) reported being abused (p< 0.001), and they were more abused verbally (63.5%) and physically (10.4%) compared to other medical sectors (p <0.001). In addition, their job performance was significantly affected (p = 0.003), with 281 (78.9%) confirming negative effect of abuse on their job performance, 307 (86.2%) feeling unsafe at their workplace (p< 0.001), and 313 (87.9%) not being satisfied with the way the administrators deal with the violence incidents at your workplace (p = 0.002). Doctors working at military hospitals had significantly higher rates of satisfaction with local policy against workplace violence at their workplace (p< 0.001), with 73 (52.5%) being satisfied with current local policy at their institution [**Table 4**]. The sample included wide variety of postgraduate programs and medical specialties, the most common to report occupational violence were general physicians (n = 266, 27.5%), followed by general surgeons (n = 113, 11.7%), internal medicine doctors (n = 111, 11:5%) and emergency physicians (n = 104, 10.7%).

**Table 4:** Perception of doctors regarding local policies related to violence against doctors.

Characteristic		Participants with history of abuse in the last 12 months (n = 611)	Participants with no history of abuse in the last 12 months (n = 358)	Total [n (%)]	P- value
**Whom do they think is more abused**	Females	80 (13.1)	58 (16.2)	138 (14.2)	0.229
	Males	243 (39.8)	149 (41.6)	392 (40.5)	
	No difference	288 (47.1)	151 (42.2)	439 (45.3)	
**Procedures for the reporting of violence at their workplace**		269 (44)	204 (57)	473 (48.8)	<0.001
**Local policy against workplace violence at their workplace**		209 (34.2)	165 (46.1)	374 (38.6)	<0.001
**Satisfied with the way the administrators deal with the violence incidents at your workplace**		67 (11)	87 (24.3)	154 (15.9)	<0.001
**Action taken to investigate the causes of the incident**		42 (6.9)	38 (10.6)	80 (8.3)	<0.001
**Believe they are protected by law**		57 (9.3)	72 (20.1)	129 (13.3)	<0.001

values are presented as number (percent).

Among those who were able to identify a main abuser, we found that 144 (14.9%) of doctors included in this study were abused mainly by the patient, 433 (44.7%) by the relatives of the patient, and 42 (4.3%) were abused by their co-workers. Doctors working at governmental medical centers were more commonly abused by the patients (19.1%) (p = 0.012), and by the relatives of the patient (55.6%) (p < 0.001), while university hospitals had the least rate (34.1%) of abuse from patients relatives (p < 0.001). No significant difference was found between males and females in the rate of being abused by the patient (p = 0.157), yet 51.5% of males were abused by the relatives of their patients (p < 0.001) compared to 32.1% of female doctors. On the other hand, 8.2% of female doctors were abused by their co-workers, compared to 2.2% of the male doctors (p < 0.001). However, abuse by co-worker was not related to the workplace (p = 0.290).

**Fig 1:**
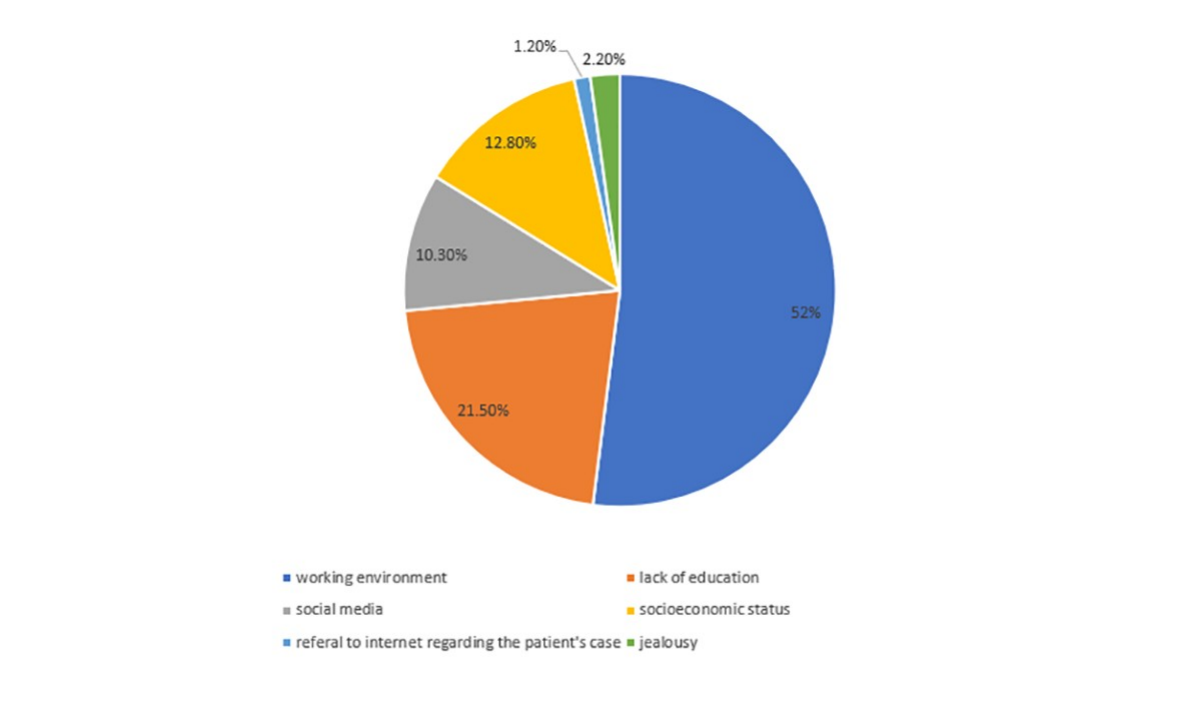
Causes of violence according to doctors’ opinions.

Upon investigating how worried doctors are regarding violence at their workplace from 1 to 5, the mean score was 3.1 ± 1.3. The 70 (7.2%) doctors who were injured due to abuse during their working time had higher scores (4 ± 1.1) than those who were not injured (p< 0.001), moreover, males scored 3.2 ± 1.3 compared to 2.9 ± 1.2 for females (p< 0.001). Pearson's r did not show a significant correlation between the aforementioned score and age of the participants (r = 0.05, p = 0.118). Those who were satisfied with the way the administrators deal with the violence incidents at their workplace had significantly lower scores (2.4 ± 1.2) (p< 0.001), and those who think they are protected by law had also lower scores (2.7 ± 1.2) (p< 0.001). **Fig 1** shows the possible causes of violence according to doctors’ opinions.

We applied multivariable regression analysis to investigate for predictors of abuse in the last 12 months. The overall model was significant (p< 0.001) **[Table 5]**. Not working on shifts (OR = 0.389; 95% C.I.: 0.188 to 0.804; p = 0.011) was negatively associated with violence incidents. Moreover, working on day shifts (OR = 0.001; 95% C.I.: <0.001 to 0.004; p<0.001) was also negatively associated with violence incidents.

**Table 5:** Multivariable regression analysis for predictors of violence against doctors.

		Value	OR	95% C.I. for OR		p-value
				Lower	Upper	
**Step 1^a^**	age	30.1 ± 6.4	0.943	0.882	1.008	0.086
	gender (Female)	340 (35.1)	1.192	0.565	2.512	0.645
	nationality (Jordanian)	924 (95.4)	0.680	0.143	3.228	0.628
	workplace					0.065
	workplace: Governmental	356 (36.7)	1.167	0.459	2.965	0.746
	workplace: Military	139 (14.3)	0.401	0.146	1.103	0.077
	workplace: Private	198 (20.4)	0.568	0.210	1.535	0.265
	work					0.353
	work (Consultant)	43 (4.4)	2.222	0.379	13.029	0.376
	work (general practitioner)	204 (21.1)	0.779	0.268	2.263	0.646
	work (intern)	167 (17.2)	1.126	0.272	4.664	0.870
	work (resident)	439 (45.3)	1.762	0.653	4.755	0.263
	shifts (No)	305 (31.5)	0.389	0.188	0.804	0.011
	timing (Day shift)	350 (36.1)	0.001	0.000	0.004	0.000
	Constant		119.813			0.002

a. Variable(s) entered on step 1: age, gender, nationality, workplace, work, shifts, timing.

b. The variables University and specialist were reference level variables for workplace and job description respectively in the dummy coding.

c. Values are presented as mean ± standard deviation and number (percent).

## Discussion

Middle East region is reported to have the highest rates of healthcare workers exposure to violence worldwide, with a prevalence of 61.3%, compared to 38.3% in Europe [24]. In Jordan, our study showed that 63.1% of doctors were exposed to at least one form of violence during the last year, which is a little higher than the average in the Middle East. This percentage is a little lower than in nurses, where there were three studies in Jordan with a prevalence of 68.2% [25], 63.9% [26] and 91.4% [27], respectively. Concerning doctors in the Middle East, there were two studies, one held in Kuwait and showed that 86% out of 101 of participating doctors have experienced verbal violence [28], and another one held in Turkey on 597 doctors and showed a very close percentage, 86.4% [29]. One strength of our study is that it included all four medical sectors, unlike Atan et al. [29], who included only university hospitals. Moreover, all hospitals from those sectors were included regardless of their capacity, and a minimum experience of 6 months was a requirement for inclusion of physicians in the this study, compared to Darawad et al, who included only centers with a capacity of more than 300 beds, and nurses with a minimum experience of 3 months only [26].

Governmental hospitals, especially during night shifts, recorded highest rates of violence incidents against doctors. In context, this can be attributed to multiple factors that potentiate violence risk factors. Although low socioeconomic status is labeled as a risk factor for violence [30], we believe that in the case of governmental hospitals in Jordan, poorly equipped facilities and surplus numbers of patients benefitting from governmental healthcare are the main reasons.

Many workplace conditions might increase violence incidents. In public hospitals, most related factors were long waiting times and overcrowding of triaging system [31]. Misconceptions about treatment strategies and staff behaviors which can cause the false perception of staff carelessness and poor communication are major reasons behind the violent attitudes of patients [32]. Other related factors in developing countries were absence of effective deterrent policies, inappropriate management of violence incidents, lack of social awareness, lack of security members or inexperienced security staff [25]. In emergency departments, the prejudices of patients or their relatives about the real emergency status of the patient can make them take aggressive attitudes against healthcare providers [33].

Violence against healthcare providers can have many negative impacts on their physical and mental health, which will eventually decrease their productivity and increase turnover intentions. For instance, in the current study, 71.8% reported that their job performance was affected, and several work changes were related to violence incidents, such as taking a leave, absence transfer to another location, and losing their job. Thus, violence can also disturb their social life, memories and thoughts, and keep them exhausted from super alertness [34]. It might also affect their job performance dramatically [35]. The lack of clear antiviolence policies in the developing countries makes healthcare workers feel lack of safety in their working environment [36]. In the current investigation, the lack of reporting procedures and local policy against workplace violence adversely affected physicians’ satisfaction with administrations’ anti-violence policies and the belief that they are protected by law. Anxiety, depression, reduced self-esteem, fear, stress, increased burnout, difficulty in interpersonal relationships and physical injuries were all reported in healthcare workers as consequences to violence exposure [37]. The impact of violence on one healthcare worker can spread its consequences on organizational level, where staff performance, productivity and team work spirit are all inevitably affected [38]. Despite all these consequences, exposure to violence in occupational environment is underreported, as shown in the current investigation. This followed the same trend as in other developing countries [39]. A fear of retaliation, stigma, lack of confidence in penalties towards the abuser, as well as consideration of violence as an expected behavior were all stated as reasons for lack of reporting [40].

In this study, male doctors were more exposed to various types of violence, this can be regarded to cultural relics and multiple laws that intensify legal penalties against women abusers in Jordan. This matches the finding in other Middle East countries like Saudi Arabia [9], Egypt [41] and turkey [42]. From this background, we can relate the tendency of doctors in Jordan to believe that males are more prone to violence than females, and that males feel more worried and tense in their working environments. Interestingly, both male and female doctors reported the same percentage of exposure to violence from patients, while relatives contributed to the noted difference in the total percentages.

The types of violence in order of frequency were verbal, emotional, physical and sexual. Sexual abuse was reported only in female doctors. The most worrisome type with a significant frequency is physical violence, where 70 doctors (7.2%) were physically injured, of which 14 doctors needed medical treatment for the injury. A truly dangerous aspect revealed by this study was that 6 doctors (0.6%) reported the use of weapons. In Jordan, there were 106 reported cases of physical violence against doctors in the years 2016–2017 [43].

Emotional abuse is any type of abuse that causes the indirect exposure of the doctor to a behavior that may result in psychological trauma, depression or posttraumatic stress disorder [44]. The unfamiliarity of the concept or the false perception of this phenomenon as a nonviolent behavior may have caused the lower percentage of reporting it.

Occupational violence has many detrimental consequences on health care system on a country-based level. Most of the doctors enrolled in our study reported anger, frustration, stress, fear, depression and headache. The most serious consequences reported were difficulty in sleeping and suicidal thoughts. Suicidal ideation is considered a psychological emergency [45]. In a two-sided doctor-patient relationship, these ideations can be catastrophic [46]. All of the aforementioned consequences directly affect the decision making ability of the doctor which is reflected on multiple patients being treated under his/her care [47].

In regard to response to violence, most of the doctors exhibited no actions, minimal percentage exhibited verbal or physical defense reactions. 119 doctors of those who were exposed to violence needed treatment (either self-employed formulas, physical or psychological). This establishes a bigger dilemma and distributes common shared feelings of lack of safety, where the doctor becomes a patient at his own working environment. This adds to the load of dangerous consequences mentioned previously. Doctors feeling of unsafety might extend to involve the doctor’s family, where 190 doctors stated the threatening of their friends and family. Furthermore, most of the doctors think that their job performance is affected. 92 doctors had to take a leave or change their working locations due to consequences related to violence and 12 doctors lost their jobs due to circumstances related to violent incidents.

Relatives were the main source of violence incidents in Jordan. The strong familial and tribal bonds in Jordan might become a negative factor when it comes to the privacy of the patient. The insistence of relatives to be involved in healthcare plans might raise a psychosocial debate and endanger the confidentiality of patients’ information, which should be protected by doctors. Moreover, even though the most common source of violence was the patient and his/her relatives [48], violence can come from coworkers in the same occupational environment [49], for which law protection must be implemented and well established for doctors and other healthcare provider. One of the major points brought up by this study was that 120 doctors felt protected by law, yet only 80 took legal actions. The reluctancy of doctors to take legal actions against violence incidents can encourage such behaviors. Another view of the subject was that legal actions might provoke intentions of violence which can lead to more harmful consequences. In the current study, only 38.6% doctors believe they have local policy to deal with violence at their workplace, and less than 20% of doctors in governmental hospitals are convinced that local policies protect them. Nevertheless, this was not the case in military hospitals, where more than 50% of doctors think they are protected. We can relate this phenomenon to the better familiarity of military doctors with the policies and rules, where acquiring knowledge about the policies is a compulsory requirement. This can explain why 52.5% of military doctors were satisfied by their administrative actions. Doctors who were satisfied with the way the administrators dealt with the violence incidents at their workplace had significantly lower scores of feeling worried at work, and those who think they are protected by law also had lower scores.

A study conducted in Jordan in 2016 regarding the policies countering occupational violence identified many problems in the sociocultural and economical contexts that were not valued by the policies [50]. In addition, multiple erroneous modifications to the law were made that led directly to the incremental figures of violence against physicians in Jordan. Action plan was suggested and urged to be taken, but not yet approved.

The main limitation of this study is that it is a cross-sectional questionnaire-based investigation, rather than being a registry for cases of violence acts against physicians. We recommend future studies to examine workplace violence against physicians and all healthcare workers using an online registry or database that allows investigating these incidents. Moreover, we did not investigate patient-related factors and their relationship with violence against physicians. Several factors can be investigated in order to have a better understanding of factors related to this behavior, such as waiting times, physicians’ departments, triage system efficacy at their departments, staff and patient’s behavior, demographic factors of the patients, and their psychological wellbeing [51].

## Conclusion and recommendations

In conclusion, the study highlighted the higher rate of violence against physicians in the governmental sector, in addition to the negative effect of abuse on their performance. Moreover, male physicians had higher incidence of workplace abuse in the last one year. Strategies that ease and promote the real application of anti-violence policies should become our future target. Policies that might personalize the incidents and push physicians to personal confrontations with abusers should be discouraged. Awareness campaigns and staff training programs on the management and prevention of occupational violence are essential in order to improve job performance and the quality of care delivered by physicians at different medical sectors.

## Supporting information

S1 File Research questionnaire. (PDF)

S2 File Research data. (XLSX)
