# Supplementary material for: Violence against physicians in Jordan: An analytical cross-sectional study
Source: PLoS One. 2021 Jan 25;16(1):e0245192. doi: 10.1371/journal.pone.0245192 (PMC7833172; doi:10.1371/journal.pone.0245192)
Supplement: S1 File — (PDF) [file pone.0245192.s001.pdf]

Prevalence of violence against doctors in Jordan

* Required

1. **Age** *

2. **gender** *

Mark only one oval.

male

female

3. **work assignment** *

Mark only one oval.

medical student

intern

General physician

resident

consultant استشاري

specialist اخصائي

4. **work place** *

Mark only one oval.

goverment

university hospital

military

private

5. **nationality** *

Mark only one oval.

jordanian

non jordanian

6. **specialty** *

Mark only one oval.

internal medicine

general surgery

obstetrics and gynecology

pediatrics

orthopedics

emergency

dentist

General physician

Other:

7. **do you work shifts** *

Mark only one oval.

Yes

No

8. **The patients you most frequently work with are** *

Check all that apply.

Children

Adolescents and adults

Elderly

violence characteristics

9. **Have you been abused before in the last 12 months? (if NO go to next section)** *

Mark only one oval.

Yes

No

10. **if yes how many times did you experience violence during that period?**

Mark only one oval.

once

2-3

more than 3

11. **If yes What was the abuse type?**

Mark only one oval.

physical

verbal

emotional

sexual

Other:

12. **If the abuse was physical, was it with using a weapon?**

Mark only one oval.

Yes

No

13. **if it was sexual abuse , was this reported?**

Mark only one oval.

Yes

No

14. **Who was the abuser?**

Mark only one oval.

The patient

relatives of patient

Co-worker

Other:

15. **where was the place of the assault**

Mark only one oval.

Emergency department

Outpatient clinic

Intensive care units (icu/ccu/nicu/picu)

Inpatient department - floor

operating rooms

Other:

16. **was the abuse reported?**

Mark only one oval.

Yes

No

17. **How did you respond to the incident?**

Check all that apply.

took no action

tried to defend myself physically

told friends/family

sought counselling

told a colleague

reported it to a senior staff member

sought help from the union ((النقابة

completed incident/accident form

pursued prosecution ((القضاء

Other:

18. **at what period of the day was the abuse?**

Mark only one oval.

day shift

night shift

19. **Which day of the week did it happen?**

Check all that apply.

week days

week end

violence consequences

20. **Were you injured as a result of the violent incident?**

Mark only one oval.

Yes

No

21. **what emotions did you experience as a consequence of violence ?**

Check all that apply.

hedache /fatigue

fear /stress

anger / frustration

depression

irritability

difficulty sleeping

Other:

22. **did you require formal treatment for the injuries?**

Mark only one oval.

no treatment

physician (treated for an injury)

i treated myself

psyciatric

23. **Did your family or friends have been threatened because of violence?** *

Mark only one oval.

Yes

No

24. **Does abuse affect your job performance?** *

Mark only one oval.

Yes

No

violence and work

25. **Did you feel unsafe in workplace because of abuse ?** *

Mark only one oval.

Yes

No

26. **How worried are you about violence in your current workplace? (Please rate: 1 = not worried at all; 5 = very worried)** *

Mark only one oval.

1 2 3 4 5

27. **what work changes did occur as a result of violence?** *

Mark only one oval.

nothing

leave or abcence

lost my job

transfer to another location

28. **Whom do you think is more abused?** *

Mark only one oval.

male

Female

No difference

29. **Are there procedures for the reporting of violence in your workplace?** *

Mark only one oval.

Yes

No

30. **do you have a local policy against workplace violence in your workplace?** *

Mark only one oval.

Yes

No

31. **are you satisfied with the way the administrators deals with the violence incidents at your**
workplace ? *

Mark only one oval.

Yes

No

32. **Was any action taken to investigate the causes of the incident?**

Mark only one oval.

Yes

No

don't know

33. **as a health care worker, do you think you are protected by law ?** *

Mark only one oval.

Yes

No

34. **in your opinion, what is the cause of violence against doctors?** *

Mark only one oval.

decreased awareness of medical problems

the internet use for the cause of patinet's illness

socioeconomic causes (poverty for example)

social media

jealousy

bad work environment (بيئة العمل مثل عدم توفر بعض الخدمات او نقص عدد الاطباء او مشاكل اخرى لها علاقة ببيئة العمل)
